# Does your group matter? How group function impacts educational outcomes in problem-based learning: a scoping review

**DOI:** 10.1186/s12909-022-03966-8

**Published:** 2022-12-29

**Authors:** Athena Li, Elif Bilgic, Amy Keuhl, Matthew Sibbald

**Affiliations:** 1 Bachelor of Health Sciences (Honours), McMaster University, Hamilton, Canada; 2 Department of Pediatrics, McMaster University, Hamilton, Canada; 3 McMaster Education Research, Innovation and Theory (MERIT) Program, McMaster University, Hamilton, Canada; 4 Department of Medicine, McMaster University, Hamilton, Canada

**Keywords:** Cognitive processing, Curriculum design, Education, Learning outcome, Problem-based learning

## Abstract

**Background:**

Problem-based learning (PBL) is a common instructional method in undergraduate health professions training. Group interactions with and within PBL curricula may influence learning outcomes, yet few studies have synthesized the existing evidence. This scoping review summarized the literature examining the influence of group function on individual student PBL outcomes. Following Kirkpatrick’s framework, experiential, academic, and behavioral outcomes were considered. The impacts of three aspects of group function were explored: (1) *Group Composition* (identities and diversity), (2) *Group Processes* (conduct and climate, motivation and confidence, and facilitation), and (3) *PBL Processes* (tutorial activities).

**Methods:**

A literature search was conducted using Medline, CINAHL, and APA PsychInfo from 1980–2021, with the help of a librarian. English-language empirical studies and reviews that related group function to learning outcome, as defined, in undergraduate health professions PBL curricula were included. Relevant references from included articles were also added if eligibility criteria were met. The methods, results, discussions, and limitations of the sample were summarized narratively.

**Results:**

The final sample (*n* = 48) varied greatly in context, design, and results. Most studies examined junior medical students (*n* = 32), used questionnaires for data collection (*n* = 29), and reported immediate cross-sectional outcomes (*n* = 34). *Group Processes* was the most frequently examined aspect of group function (*n* = 29), followed by *Group Composition* (*n* = 26) and *PBL Processes* (*n* = 12). The relationships between group function and outcomes were not consistent across studies. PBL experiences were generally highly rated, but favorable student experiences were not reliable indicators of better academic or behavioral outcomes. Conversely, problematic group behaviors were not predictors of poorer grades. Common confounders of outcome measurements included exam pressure and self-study.

**Conclusions:**

The main findings of the review suggested that (1) group function is more predictive of experiential than academic or behavioral PBL outcomes, and (2) different Kirkpatrick levels of outcomes are not highly correlated to each other. More research is needed to understand the complexity of group function in PBL tutorials under variable study contexts and better inform curricular training and design. Standardized tools for measuring PBL group function may be required for more conclusive findings.

**Supplementary Information:**

The online version contains supplementary material available at 10.1186/s12909-022-03966-8.

## Introduction

Problem-based learning (PBL) is a method of teaching and learning that involves solving case problems in small groups. Built upon the philosophical foundation that knowledge is socially co-constructed, this pedagogy depends on self-directed learning, group discussion, problem-solving, and peer teaching as its primary processes [1]. Conceivably, group function is the backbone of this educational format. Since its inception in the late 1960’s at McMaster University [1], PBL has evolved and expanded on a global scale. Today, it finds its place as a central component of health professions education worldwide [2, 3]. Given the centrality and ubiquity of PBL in the training of health professionals, the practical implications of understanding its ability to produce individual student learning outcomes is of greater importance than ever before [4].

In a typical PBL tutorial, students are divided into small groups and presented with a relevant problem, most commonly in the form of a clinical case or problem [5]. The groups are tasked with identifying learning issues and objectives and formulating hypotheses about the case, then sent off for independent study on the topic [5]. Students later bring their findings back to the group and collaboratively synthesize knowledge through various cognitive processes that are evoked by group discussion [5, 6]. Courses tend to conclude with an assessment and reflection of group learning, with regard to both knowledge content and group process. A facilitator, also known as a tutor, is present throughout to guide tutorial activities [5]. At many institutions, tutors and/or students also undergo training prior to the start of a semester specifically to learn how to engage in a PBL setting [7], though the degrees of training and training satisfaction are variable [8]. The exact format of PBL also differs across institutions and programs [9]. Variations may involve different group sizes and compositions, different tutor identities, expertise, and roles [10], the presence or absence of individual and group assessments during various stages of the process, and additional components such as presenting small-group findings to a larger class [9].

**Fig. 1:**
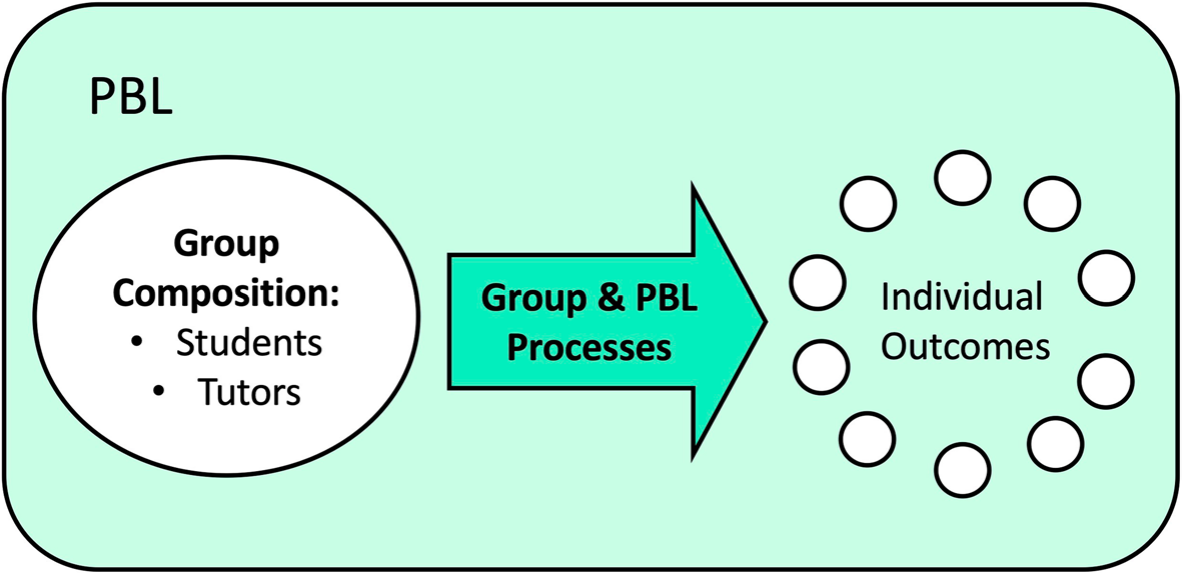
Conceptualization of group, PBL, and outcomes

Given its collaborative nature, PBL uniquely provides the opportunity to practice skills required for collective learning, such as the discussion and expansion of ideas, critical reflection, cooperation, communication with group members, and responding to the social influences of the group [11]. However, the inherent social dimension of groupwork introduces challenges [12] where students and groups may differ in academic skills, level of contribution, tolerance of other students, social status, etc [13]. Apart from the students, the tutor is also inherently involved in the functioning of the group. Tutors may contribute to both social functions, such as guiding the learning climate, and learning functions, such as planning tutorial activities, facilitating group processes, and evaluating outcomes [10]. The effectiveness or ineffectiveness of PBL towards individual learning outcomes may thus be conceptually dependent upon the abilities of the students and tutors to engage in group and curricular processes (Fig. 1) [12, 14].

The literature debates whether the merits of group learning are realized in standard practice [9, 15, 16]. Currently, there is no accessible knowledge base or tool to help researchers and educators understand the value of group function in contributing to student learning outcomes. Literature reviews on this topic are few, none of which focus on group function comprehensively in an undergraduate health professions population. It is currently unclear what aspects of group function specifically impact learning outcomes, to what degree, and in what manner. A new scoping review is thus valuable to help researchers understand the range of work that has been done so far on this topic and identify areas in need of further investigation, as well as help educators inform curriculum and training design.

### Definitions

#### PBL

PBL has a broad range of definitions that span from the ideologic to the pedagogic. Several models of PBL have been identified, such as the Aalborg model, the Maastricht ‘Seven-Step’ model, and project-based PBL, which differ in details such as the frequency of attendance of the tutor at tutorials, the number of problems presented, and the level of tutorial structure [17]. Hung also conceptualized a nuanced PBL step-ladder that points from hybrid curricula with PBL components to pure PBL, based on the two dimensions of structuredness of cases and self-directedness of tutorials [9]. It has also been pointed out that the definition of PBL often manifests differently in theory than in practice, the latter being dependent on tutors’ and students’ human abilities to act out PBL in a real-world setting [9]. In recognizing the complications of PBL theory and implementation, this review considers the definition of PBL in its broadest sense and includes any format of learning that incorporates core PBL components (i.e. students work in groups and undergo self-directed learning to solve a clinical case or problem in the presence of a tutor [2]), within or without other curricular components.

PBL may also be differentiated from related pedagogy, such as team-based learning (TBL), small-group learning (SGL), and case-based learning (CBL), which are distinct in their origins, features, and goals, and yet may share overlapping elements [18, 19]. In some cases, the use of pedogeological terminologies have been blurred in practice across institutions and in research [9, 18, 19]. This review thus also considered literature using TBL or SGL terminology, on a case-by-case basis, so long as the curricular processes resemble those described above.

#### Group and group function

The PBL group is defined, according to Barrows’ model, as consisting of a number of students and a tutor, who engage collaboratively to solve tutorial problems [20]. Group function, then, refers to the processes of interactivity between the participants of the group, and between the participants and the PBL curriculum.

#### Learning outcome

Learning outcomes may be organized in several ways. For instance, PBL is recognized for its impact on both knowledge learning and social outcomes, at an individual and group level [11]. This review focuses on individual student learning outcomes, which most directly aligns with the qualifications of an independent health professional.

### Research objective

The objective of this scoping review is to explore the breadth and depth of the existing literature that relate group function to PBL outcomes, to identify aspects of group function that are important in contributing to individual student learning outcomes in undergraduate health professional PBL**.**

## Methods

This scoping review follows the PRISMA checklist for Transparent Reporting of Systematic Reviews and the JBI methodology for scoping reviews [21].

### Search strategy

A preliminary search in Medline, CINAHL, and APA PsychInfo was conducted in October 2021 to identify relevant articles and inform the search strategy. The research question was broken down into search terms in natural language, which was then used to develop a full search strategy with the help of a librarian at McMaster Health Sciences Library. Search terms were adapted for each database, mapped to subject heading, and used as keywords to search titles and abstracts. Search strategy details are provided (Appendix 1).

### Inclusion criteria

Included studies (1) are in English, (2) are published no earlier than 1980, out of consideration for practicality and relevance to the modern social context, (3) are empirical studies using any methodology or systematic or scoping reviews, (4) studied an undergraduate healthcare student population, and (5) explored aspects of group function, as defined, in relation to student learning outcome in a PBL setting.

Opinion texts, unpublished literature, and experimental studies that took place within simulated environments were excluded. Studies that aimed to compare PBL outcomes to traditional learning outcomes were also excluded, as the aim of this review is to understand the role of group components within PBL, not to distinguish the merits of PBL from other learning contexts.

### Study screening and inclusion

All identified citations were uploaded into the Covidence tool for removal of duplicate records and primary screening. A pilot test of the inclusion criteria was conducted on a small sample of articles by two independent reviewers. The inclusion criteria were revised for clarity after discussion. Primary screening of titles and abstracts by the two reviewers took place between October and December. 73 inter-reviewer conflicts were encountered, such as when there was a lack of clarity about the relevance to group function or the population being studied in the abstract. These were resolved by discussion, over two online meetings. By mutual agreement, irrelevant abstracts were excluded, and truly ambiguous abstracts were conservatively included for full text review.

The first author conducted a full text review for abstracts labeled “yes” or “maybe” for inclusion by both reviewers. Eligible articles were included in the analysis. Additional relevant articles were identified by back-searching the reference section of included studies. These were included in the analysis if inclusion criteria were met after full text screening.

### Data extraction

A data extraction sheet, developed by the first author, was used to extract and organize details from the included full text sample. Articles were reviewed for publication details, participants, PBL context, aims/purpose, methods, relevant key findings, and limitations. Additional fields were added and revisited as data extraction proceeded. A final draft of the extraction form is provided (Appendix 2).

### Quality appraisal

A basic appraisal was also conducted to inform readers of the methodological quality of the included articles (Appendix 3). Quantitative and mixed-methods studies were assessed using the Medical Education Research Study Quality Instrument (MERSQI) framework [22]. Qualitative studies were assessed using a 12-item grid by Côté and Turgeon [23]. These models were selected for their particular focus on medical education research. Scoping and systematic reviews were assessed using the Joanna Briggs Institute Critical Appraisal Checklist for Systematic Reviews and Research Syntheses [24]. Quality appraisal scores did not influence screening decisions.

### Data analysis framework

#### Aspects of group function

Fonteijn and Dolmans previously identified several variables that affect group functioning in PBL and categorized these into three clusters [25]. The first, "the resource pool”, considers the intrinsic properties of the group, such as group size and individual differences in identity, ability, and experience. Next, “the learning task and group learning processes” includes both the quality of the tutorial problem and concepts of autonomy, group climate, and team learning behaviors that enable the co-construction of knowledge. Third, “the learning context” broadly refers to the situational factors that influence how groups may interact, including the discipline of study, cultural context, socialization and training of students, and role of the tutor [25].

To practically aggregate data, this review gains inspiration from Fonteijn and Dolmans [25] but considers tutor components as a human part of the group rather than as a part of the curricular context. As such, group function will be organized into three aspects: *Group Composition, Group Processes, and PBL Processes. Group Composition* aligns closely with “the resource pool” and includes tutor identities in addition to student identities and other human resource factors. *Group Processes* reflects the behavioral and cognitive dimensions of “group learning processes” above, including student conduct and social climate, and motivation and confidence. *Group Processes* additionally considers the role of tutor facilitation. Finally, “the learning task” is reworked into *PBL Processes*, which examines factors related to tutorial activities, such as case quality and feedback.

#### Levels of learning outcome

The Kirkpatrick (KP) Model is a recognized method of assessing the efficacy of education and professional training programs from a learner-focused perspective [26]. This model, developed by Donald Kirkpatrick in the 1950s, is valuable in its ability to provide a simple categorization of educational outcomes in four ascending levels [26, 27]. In the context of health professions PBL, these levels are: i) KP1, which refers to students’ immediate reaction to a training program, including their perceptions and experience of PBL, ii) KP2, which refers to the learning of information, is measured by indicators of academic performance, iii) KP3, which refers to the impact on students’ behavior, including leadership and conduct, and iv) KP4, which refers to long term professional results in the workplace [28].

A previous systematic review on the effectiveness of TBL by Fatmi et al. interestingly pointed out a lack of correlation between KP1 and higher level outcomes [29], suggesting the importance of examining each level of KP outcomes separately.

#### Summarizing and reporting findings

Included articles were characterized numerically for year of publication, studied population and geographical distribution, study methods, aspects of group function studied, and KP level of outcomes reported. Overall results pertaining to the objective of this review were organized by aspects of group function and summarized narratively.

## Results

### Literature scope and characteristics

**Fig. 2:**
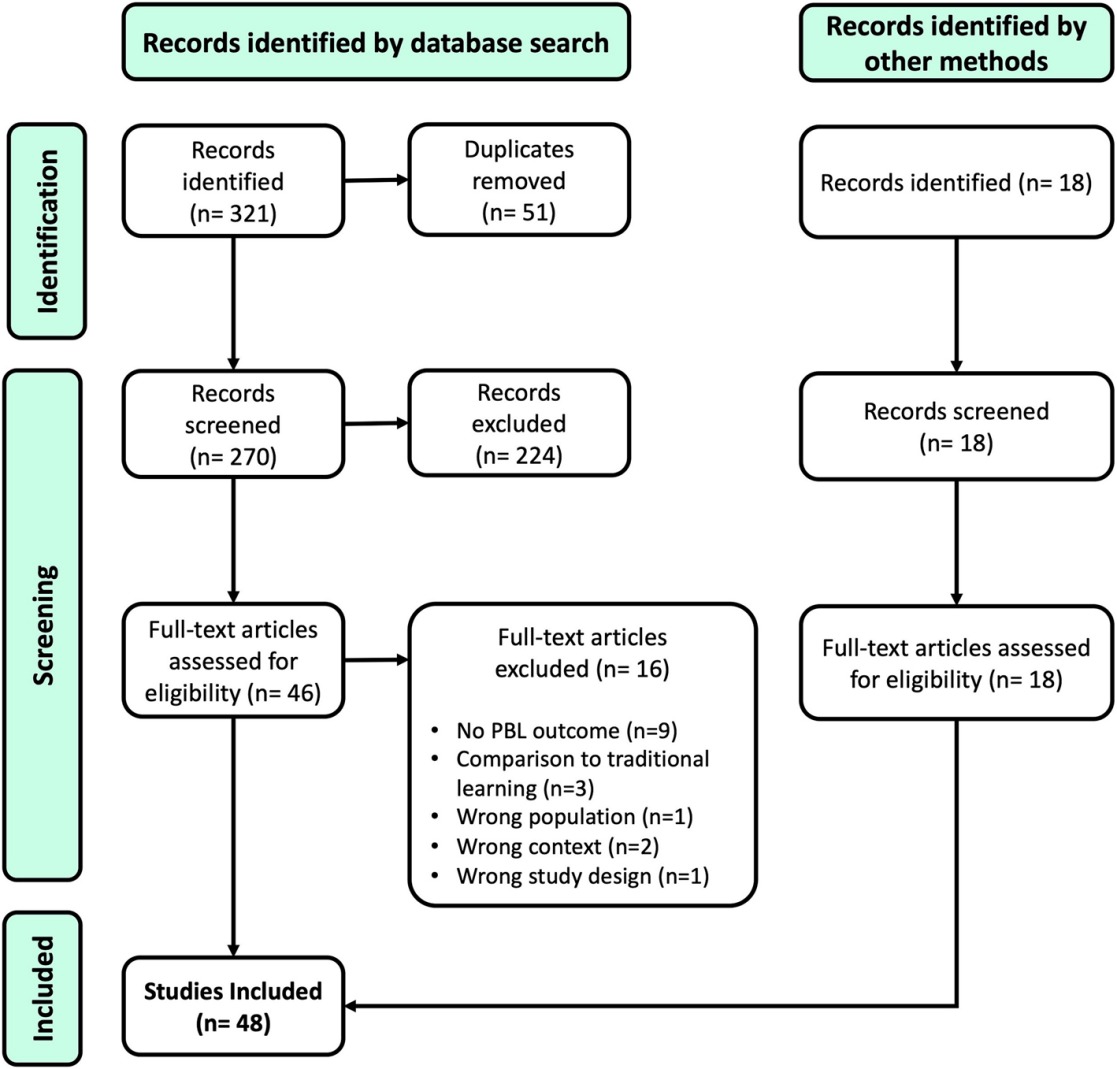
PRISMA-ScR Flow Diagram for study selection and inclusion

Results of the initial search, inclusion process, and exclusion details for ineligible full text articles are reported in Fig. 2. The final sample consisted of 48 articles [30–77].

The final sample of studies (*n* = 48) was highly varied in context, design, and outcomes. Details are provided in Table 1. The search results yielded studies published between 1992 and 2020.

**Table 1:** Study characteristics

Study Characteristics (Total *n* = 48)	n	Study Characteristics (Total *n* = 48)	n
**Year of Publication**		**Studied Population**^**a**^	
1992–2000	6	Medicine	32
2001–2010	21	Nursing	3
2011–2020	21	Health Sciences	5
**Study Methods**		Medicine and Dentistry	2
Quantitative	31	Tutors Only	1
Qualitative	8	Medical Students and Tutors	5
Mixed Methods	7	Students' Year of Study	
Literature Review	2	Year 1	12
Study Design		Year 2	8
Questionnaire/Survey	29	Year 3	2
RCT	6	Year 4	3
Interview/Focus Group	4	Years 1 and 2	10
Observational	4	Years 1–3	2
Pretest–Posttest	3	Years 1–4	4
Scoping Review	1	Years 2–4	1
Systematic Review	1	"Junior"	1
Study Duration		Unspecified or N/A	5
Cross Sectional	39	Country of Educational Institution	
Retrospective on a Specific PBL Session, Module, Year, etc	34	Netherlands	11
		United States of America (USA)	8
Retrospective on General Experience	5	Australia	5
Longitudinal	7	Canada	4
N/A	2	United Kingdom (UK)	2
**Count of Aspects of Group Function**^b^		Finland	2
Group Composition	26	United Arab Emirates (UAE)	2
Group Processes	29	Bahrain	2
PBL Processes	12	Indonesia	2
**Count of KP Levels of Outcome Studied**^b^		Singapore	2
KP 1	38	Ireland	1
KP 2	24	Lebanon	1
KP 3	6	Iran	1
		Korea	1
		Japan	1
		China	1

^a^Population is students unless otherwise specified

^b^The sum of the tally exceeds the sample size (*n* = 48) as many articles touched upon results related to more than one aspect of group function and more than one KP level of student outcome

### Studied population and geographical distribution

Most studies focused on medical students (*n* = 32). Others examined a mixture of programs including nursing, medicine and dentistry, and undergraduate health sciences, or a mixture of student and tutor populations. Geographically, there was representation across Europe (*n* = 16), North America (*n* = 12), East Asia (*n* = 7), the Middle East (*n* = 6), and Australia (*n* = 5).

### Study methods

Two articles were systematic or scoping reviews. The remaining 46 articles were empirical studies, reliant on quantitative (*n* = 31), qualitative (*n* = 8), or mixed (*n* = 7) methods to evaluate various group functions and learning outcomes. Field study questionnaires using Likert-type scales were the most common study design (*n* = 29), followed by randomized control trials (RCTs) (*n* = 6), semi-structured interviews (*n* = 4), and observational studies (*n* = 4). Reported outcomes were largely cross-sectional (*n* = 39), and longitudinal studies did not exceed two years in length of follow-up.

### Aspects of group function studied

For ease of categorization, a count of mentions of each aspect of group function is tallied in Table 1. The greatest number of articles examined elements related to *Group Processes* (n = 29), and the least number touched on *PBL Processes* (*n* = 12).

### Learning outcomes

Most frequently reported outcomes were student experiences or perceptions of learning (KP1) (*n* = 38), followed by knowledge acquisition (KP 2) (*n* = 24). Very few studies reported behavioral outcomes (KP3) (*n* = 6), and no studies followed KP4 outcomes into the workplace.

Individual results of each included study are summarized in Table 2 [30–77].

**Table 2:** Study details and summary of results

Author, Year	Title	Study Design & Duration	Participant^a^: Study Yr (*n* =), Institution, Country	PBL group size	PBL context	Results		
						**Group Identity**	**Group Processes**	**PBL Processes**
Hayashi et al., 2013 [30]	Comparison of tutored group with tutorless group in problem-based mixed learning sessions: a randomized cross-matched study	RCT, quantitative, cross sectional (on one PBL session)	Yr 1 (*n* = 202), Aichi Medical University, Japan	7–8	Daily small group discussions and short lecture regarding recurring scenario (4 discussions make a PBL session); daily report on group discussions and learning details and formative tutor feedback; written exam on the last day of each PBL session; groups are switched after one PBL session	Compared faculty-tutored and tutorless PBL. Faculty-tutored students reported a more favorable learning experience. Both groups of students performed comparably on exams, but tutorless students showed greater in-group variance on exam scores	Group dynamics in both tutored and untutored conditions were inconsistently variable	
Hay & Katsikitis, 2001 [31]	The 'expert' in problem-based and case-based learning: necessary or not?	RCT, quantitative, cross sectional (on one unit)	Yr 4 (*n* = 144), Medical Faculty at University of Adelaide, Australia	10–12	PBL involves 3 weekly 90-min sessions— a brief initial scenario presentation (details given later), group synthesis of data and hypotheses, and identification of learning issues; CBL involves one 90-min session— full case presentation and supplementary reading provided in advance, small group presents to a larger group, more tutor feedback and assistance	Students did not especially prefer clinician or non-clinician tutors. Clinician-tutored groups performed better on a voluntary knowledge test, while the non-clinician tutor was perceived to be better at group management and communication skills		
Shields et al., 2007 [32]	A faculty development program to train tutors to be discussion leaders rather than facilitators	Pretest–posttest, quantitative, cross sectional (on one course)	Yr 2 (*n* = 508), Harvard Medical School, USA	7–9	Three 90-min PBL tutorials per week over a 3-week course; tutors are trained in asking guiding questions, summarizing major points, and using schematics; cases and tutorial objectives are provided before tutorial; External components: 1–1 tutor–student meetings for personal feedback and identification of quiet/dominant students	Expert and non-expert tutors were rated equally favorably by students. There was little difference in academic achievement. There was little difference in academic achievement	Students enjoyed the autonomy created by tutors acting as discussion leaders (by asking question, summarizing material, and creating visual schematics), rather than facilitator. Course satisfaction improved over years of study	
Davis et al., 1992 [33]	Effects of expert and non-expert facilitators on the small-group process and on student performance	Observational, mixed method, cross sectional (over one PBL session)	Yr 2 (*n* = 156), University of Michigan Medical School, USA	7–8	PBL involves problem identification, setting objectives, self-study followed by report of findings to the group	Students’ perception and exam grades were higher for expert than non-expert tutors	Expert and non-expert tutors did not systematically differ in facilitation style. Student self-direction was prominent in both tutor groups	
Schmidt et al., 1993 [34]	Influence of tutors' subject-matter expertise on student effort and achievement in problem-based learning	Questionnaire, quantitative, cross sectional (over one unit)	Yr 1–4 health science students (*n* = 1120), University of Limburg, Netherlands	10	Two 120-min PBL tutorials per week over a 6-week unit; students complete a course evaluation and achievement test at end of each unit	Part 1: Results of existing studies on the effectiveness of expert versus non expert tutors are inconclusive. Some studies found a preference for student tutors over staff tutors. Part 2: Students' exam scores and self-study time was greater for expert than non-expert tutors. The effect of tutor expertise diminishes with year of study	Expert tutors were better at identifying case objectives, while non experts were more focused on group functioning. Both are important to PBL tutoring	
Qin et al., 2010 [35]	Application of problem-based learning in a large class in stomatology course	Questionnaire, quantitative, cross sectional (on one unit)	Yr 4 (*n* = 236), China Medical University, China	7–8	Curriculum of a 30-h unit involves 12 h of lecture and 18 h of PBL (two 100-min PBL tutorials per week); both an expert and nonexpert tutor were present; groups were naturally formed and instructed to appoint a different group leader for different cases; theoretical and case analysis exam, and self- and peer-evaluations occur throughout	Students enjoyed experts and non-experts for content learning and guiding group dynamics, respectively. Students with less PBL experience preferred expert tutors. Students enjoyed PBL and performed equally well on exams regardless of previous PBL experience		
Groves et al., 2005 [36]	Tutoring in problem-based learning medical curricula: the influence of tutor background and style on effectiveness	Questionnaire, quantitative, cross sectional (over one semester)	Yr 1 students (*n*-270) and tutors (n = 50), University of Queensland, Australia	10	Three 11-week terms in a school year; groups remained consistent for the year	Clinician tutors were rated higher than non-clinicians on cognitive congruence, focus on summative tests, degree of authority, role congruence, and encouragement of group dynamics. Staff tutors were higher than non-staff tutors on the same. Older tutors were higher on all but role congruence. However, overall tutor ratings did not vary significantly		
Kassab et al., 2005 [37]	Student-led tutorials in problem-based learning: educational outcomes and students' perceptions	RCT, quantitative, cross sectional (on one unit)	Yr 3 (*n* = 91), College of Medicine and Medical Science Arabian Gulf University, Bahrain	///	unspecified	Student tutors bonded better with students and faculty tutors were rated as better at problem discussion, but tutor identity had no impact on knowledge acquisition		
Kassab et al., 2005 [38]	Gender-related differences in learning in student-led PBL tutorials	RCT, quantitative, cross sectional (on one unit)	Yr 3 (*n* = 91), College of Medicine and Medical Science Arabian Gulf University, Bahrain	///	2 PBL tutorials per week in a pre-clinical curriculum; different students are elected as peer-tutor each week; students undergo a 1-day workshop on tutoring skills	Female student tutors were rated as better at displaying professional behavior and giving feedback than male student tutors, but student tutors struggled with problem discussion and analysis regardless of gender. Tutor gender also had no impact on knowledge acquisition		
Ten Cate et al., 2012 [39]	Academic achievement of students tutored by near-peers	Questionnaire, quantitative, cross sectional (over one course)	Yr 1–3 (*n* = 9923), University Medical Center Utrecht’s Medical School, Netherlands	14 avg	2 PBL tutorials per week for each 4–6-week course	Student course grades did not differ significantly for near-peer versus faculty tutor groups		
Widyahening et al., 2019 [40]	Evaluation of the role of near-peer teaching in critical appraisal skills learning: a randomized crossover trial	RCT, quantitative, cross sectional (on one module)	Yr 4 (*n* = 241), Faculty of Medicine Universitas Indonesia	10–11	Curriculum involves 4-week modules with lectures, 120-min PBL sessions, presentations on study design/conduct, and computer labs (data search/analysis); PBL follows lectures and involves appraising journal articles on diagnosis, therapy, prognosis etc	Near-peer and faculty tutored students were comparable for content knowledge, critical appraisal ability, attitudes towards PBL, and confidence in their own skills. Near-peers were more readily accepted by students		
Chng et al., 2015 [41]	To what extent do tutor-related behaviours influence student learning in PBL?	Questionnaire, quantitative, longitudinal (over one PBL session)—two studies reported	Study 1: Yr 2 (*n* = 77) / Study 2: unspecified year of study (n = 637), Singapore Polytechnic Faculty of Science, Singapore	≥ 5	PBL cycle was completed within 1 day; phase 1 = 1 h problem analysis, phase 2 = 4 h self-directed learning w minor tutor guidance, phase 3 = 2 h reporting and peer eval; *tutors were selected based on previous congruence ratings for this study	Study 1: Tutor ratings were higher for high-congruence tutors, but student recall ability was higher for low congruence tutors. Study 2: Students with high-congruence tutors had higher module scores. This effect was consistent for high, mid, and low performance students		
Cianciolo et al., 2016 [42]	Observational analysis of near-peer and faculty tutoring in problem-based learning groups	Observational, qualitative, cross sectional (on three units)	Yr 2 (*n* = 46), Southern Illinois University School of Medicine, USA	6	2 PBL sessions per week over 9 weeks; Multiple-choice test and standardised patient exam administered on the last week; *PBL groups are stratified by demographic characteristics and academic achievement	All tutors exhibit a degree of professional congruence to students. Student tutors were particularly valued for their social congruence and empathy for learner needs	Tutor identity (Year 4 student, clinical faculty, basic science faculty) showed no significant differences in the observable nature of student behavior and group interaction. Facilitation practices have greater variation within tutor types than between	
Schmidt, 1994 [43]	Resolving inconsistencies in tutor expertise research: does lack of structure cause students to seek tutor guidance?	Questionnaire, quantitative, cross sectional (over one unit)	Unspecified health science students (*n* = 1800) and tutors (*n* = 320), University of Limburg, Netherlands	10	Two 120-min PBL tutorials per week over a 6-week unit	Tutor expertise impacted student achievement primarily in modules where students had low prior knowledge and in poorly structured modules. Student tutors produced consistent achievement scores regardless of prior knowledge and course structure		
Vasan et al., 2009 [44]	A survey of student perceptions of team-based learning in anatomy curriculum: favorable views unrelated to grades	Questionnaire, quantitative, cross sectional (on one course)	Yr 1 (*n* = 317), New Jersey Medical School, USA	8	TBL involves assigned pre-class readings, identification of learning issues, individual testing, 90-min group discussion, group testing, and peer evaluation; tutors float between groups during tutorial; curricular context involves presentations and dissection labs but no basic knowledge lectures	Perception of PBL was generally favorable, but increased with higher anticipated grades, while perception of groupwork was not significantly impacted by anticipated grades		
Gallagher, 2009 [45]	Collaborative essay testing: group work that counts	Questionnaire, mixed method, cross sectional (on one course)	Junior level nursing students (*n* = 163), Saint Xavier University of Chicago, USA	3–4	Groups are engaged in case studies, scenario reviews, etc. and present to a larger group under guidance of a faculty facilitator; 3 closed-book exams with group and individual components throughout semester; *groups are assigned	Perception of learning was irrelevant to grades	Students acknowledged the professional need for collaborative skills and perceived good group dynamics overall. Students felt more motivated to study and more confident in their knowledge in group settings	Students were in favor of group testing
Wahid et al., 2015 [46]	Students’ characteristics related to their performances in problem-based learning	Questionnaire, quantitative, cross sectional (over one module)	Yr 1–3 (*n* = 539), Faculty of Medicine Universitas Indonesia, Indonesia	///	Two 120–180 min PBL sessions per week, involving 4–5 cases over each 6-week module; PBL is supplemented by lectures, labs, conferences, etc.); module grades incorporate knowledge tests and learning process evaluation scores; *tutors are physicians or faculty with biomedical backgrounds	Academic performance did not vary by gender and geography. Performance increased with years of study and varied by admission method. Talent-scouted students (based on grades and extracurriculars) were highest performing, while students admitted based on standard exams were lowest performing		
Wimmers & Lee, 2015 [47]	Identifying longitudinal growth trajectories of learning domains in problem-based learning: a latent growth curve modeling approach using SEM	Questionnaire, quantitative, longitudinal (over two years)	Yr 1–2 (*n* = 296), UCLA School of Medicine, USA	8	2 PBL sessions a week over nine 5–8-week blocks in a school year; PBL is supplemented by lectures, labs, and clinical skills workshops	Students’ skills and abilities were highly variable. Professionalism was the most stable trait over time. Individual differences in ability persisted over the two-year study period		
Kamp et al., 2013 [48]	The effect of midterm peer feedback on student functioning in problem-based tutorials	Pretest–posttest, mixed methods, cross sectional (on one course)	Yr 2 health science students (*n* = 74), University of Maastricht, Netherlands	≤ 10	One 120-min PBL tutorials per week over 8-week courses; PBL involves case discussion, identification of learning issues, synthesis of findings; peer feedback given mid-way of course; all students received training giving peer feedback; *tutors switched each tutorial	Peer feedback was more effective in low-achievers than high-achievers for improving the quality of student contribution		Students suggested feedback would be more effective if given verbally and received in the context of well-defined group and individual goals
Ganguly et al., 2019 [49]	Association of group composition diversity and performance outcomes in a pre-clerkship team-based learning program	Questionnaire, quantitative, cross sectional (on one PBL session)	Yr 1 (*n* = 238), University of Texas Southwestern Medical School, USA	5–7	1–2 TBL sessions per course; TBL groups were stratified based on DISC personality assessment, science background, and gender balance	Students perceived gender as the most important aspect of diversity, but diversity in race/ethnicity most influenced academic performance. More racially diverse groups perceived diversity as more important for learning outcome when compared to less diverse groups		
Thompson et al., 2015 [50]	Team cohesiveness, team size and team performance in team-based learning teams	Questionnaire, quantitative, cross sectional (over one rotation)	Yr 3 (*n* = 975), across four university medical schools, USA	5–7	4 TBL sessions per 6-week clerkship rotation; course grades incorporate individual and group exam scores	Group exam scores increased with larger group size (5–7) and correlated with individual exam scores. Gender composition of groups was irrelevant		
Mpofu et al., 1998 [51]	Perceptions of group dynamics in problem-based learning sessions: a time to reflect on group issues ^b^	Questionnaire, mixed method, longitudinal (over one module)	Yr 1 (*n* = 46), FMHS United Arab Emirates University, UAE	7–8	One 2 h PBL session per week over a 140-h module; the role of group leader and scribe is rotated amongst members; PBL is supplemented by practical skills workshops, lectures, and self-study); *students separated by gender due to cultural reasons	Student perceptions differed significantly by gender. Females prioritized maximizing academic learning outcomes and males valued the opportunity to participate autonomously	Students rated communication and participation among the most important aspects of PBL, and dysfunctional group behavior and power imbalance among the least important. Dysfunctional group behavior was frequently ignored. The perceived importance of a leader role decreased over time	
Das Carlo et al., 2003 [52]	Medical student perceptions of factors affecting productivity of problem-based learning tutorial groups: does culture influence the outcome? ^b^	Questionnaire, quantitative, cross sectional (over one module)	Yr 1 (*n* = 115), FMHS United Arab Emirates University, UAE	8–10	Students undergo a 2-week orientation program to PBL processes and goals; PBL tutorials are 2 h long; tutors are rotated for each of 4 themes in a module; *students separated by gender due to cultural reasons	Female groups scored higher in motivation and productivity. Male groups displayed more disruptive behavior such as lateness and absenteeism	Motivation contributed to group productivity	
Burgess et. al., 2014 [53]	Medical students as peer tutors: a systematic review	Systematic review	n/a	n/a	n/a	Students who participated in PBL as peer tutors reported favorable experiences and immediate behavioral outcomes, though academic benefits were variable	Favorable experiences centered around recurrent themes of confidence and autonomy	
Koufogiannakis et al., 2005 [54]	Impact of librarians in first-year medical and dental student problem-based learning (PBL) groups: a controlled study	RCT, quantitative, cross sectional (on one block)	Yr 1 medical and dental students (*n* = 164), University of Alberta, Canada	9	4 PBL sessions per week over 6 week unites; all students undergo a 2 h library lab session at the start of the term; *tutors are physicians; *groups are stratified by demographic representation	The presence of a librarian facilitator during PBL made no difference in students’ attitudes, confidence in library skills, and exam performance		
Iqbal et al., 2016 [55]	Differential impact of student behaviours on group interaction and collaborative learning: medical students' and tutors' perspectives	Semi-structured interview, qualitative, retrospective on overall experience	Yr 1–2 students (n = 22) and tutors (*n* = 8), University of New South Wales Medical School, Australia	///	2 h weekly tutorials on scenario-based collaborative learning activities; grades incorporate individual and group assessments		Student behaviors may positively or negatively impact group cohesion (associated to students' experience of the group), group learning, or both. Timidness was viewed unfavorably while assertiveness was viewed favorably. Cohesion and learning may occur independently of each other	Feedback was thought to be important to both cohesion and learning
Van Berkel & Dolmans, 2006 [56]	The influence of tutoring competencies on problems, group functioning and student achievement in problem-based learning	Questionnaire, quantitative, cross sectional (on one module)	Yr 1–2 (*n* = 352 groups), University of Maastricht Medical School, Netherlands	9–10	2 PBL sessions per week	Regular feedback and evaluation correlated to group functioning	Tutors’ ability to stimulate self-directed learning correlated to perceived PBL case quality and group performance	PBL case quality correlated to group performance and individual test scores
Alizadeh et al., 2017 [57]	Uncover it, students would learn leadership from Team-Based Learning (TBL): The effect of guided reflection and feedback	Pretest–posttest, quantitative, cross sectional (on one block)	Yr 1 (*n* = 223), Tehran University Medical School, Iran	4–7	Preclinical curriculum is lecture predominant, supplemented by lab exercises, case-based discussions, and TBL		Role reflection may be important to the appreciation of group-based learning. Reflective capacity was promoted by feedback	Guided reflection and feedback had no effect on leadership behavior or team decision quality but did increase students’ acceptance of leadership and self-awareness for group roles
Kingsbury & Lymn, 2008 [58]	Problem-based learning and larger student groups: mutually exclusive or compatible concepts—a pilot study	Questionnaire, quantitative, cross sectional (on one module)	Yr 2 students (*n* = 111) and tutors (n = 8), University of Nottingham Medical School, UK	20–21	Different weekly topics explored over a 5-week summer module; cluster PBL methodology (7 cases distributed to smaller groups of 2–3 students for self-directed research, and finding are presented to the large group at the end of the week) followed by summary lecture to debrief clinical relevance of the case		Students and tutors generally reported a positive PBL experience, with minimal conflict in group dynamics. Tutors lacking in facilitation experience or training had worse perception of PBL	Tutors and students liked the use of a subgroup PBL format in a large class, with no comparator. Case difficulty impacted tutors’ but not students’ perception of learning effectiveness
Dolmans et al., 2001 [59]	Relationship of tutors' group-dynamics skills to their performance ratings in problem-based learning	Questionnaire, quantitative, cross sectional (study period unspecified)	Yr 1–4 (*n* = 75 groups), University of Maastricht Medical School, Netherlands	5–10	unspecified		Overall group dynamics were favorable, and groups' ratings of sponging behaviors were low. However, groups' ratings of the tutors' ability to facilitate group dynamics and reduce problematic behavior were also low	
Dolmans & Schmidt, 2006 [60]	What do we know about cognitive and motivational effects of small group tutorials in problem-based learning?	Scoping review	n/a	n/a	n/a		Group processes in PBL are driven by cognitive (reasoning, cognitive conflicts, collaborative knowledge construction) and motivational factors (intrinsic interest), which are beneficial to academic outcomes. The effects of group discussion are greater in those with less prior knowledge, and students who did not contribute in group discussions did equally well on tests	
Hendry et al., 2003 [61]	Group problems in problem-based learning	Questionnaire, mixed method, cross sectional (retrospective on general experience)	Yr 1–2 students (*n* = 143) and tutors (*n* = 76), University of Sydney Medical School, Australia	8–9	3 PBL tutorials per week (new case each week); tutors change per block over 9 blocks; PBL is supplemented by lectures		Quiet and dominant students, lateness, and absenteeism were perceived as frequent PBL issues by students and tutors. Poorly organized tutorials, superficial case problems, and dominant students were considered the most hindering for learning. Tutors felt prepared to deal with curriculum-related dysfunction, but neither tutors nor students are effective at resolving issues caused by student behavior. Students also identified dominant and uninterested tutors as problematic	
ODoherty et al., 2018 [62]	What can we learn from problem-based learning tutors at a graduate entry medical school? A mixed method approach	Questionnaire & focus group, mixed methods, cross sectional (on one course)	Yr 1–2 tutors (*n* = 33), University of Limerick School of Medicine, Ireland	///	*All tutors are clinicians		First year students were more likely to rely on tutors more as knowledge experts, compared to second year students. Tutors perceived dominant and timid students as a major challenge for facilitation. Experienced tutors were more confident in engaging in autonomous facilitation styles. The facilitation process is not standardized and is complicated by external factors	
Van Mook et al., 2007 [63]	Factors inhibiting assessment of students' professional behaviour in the tutorial group during problem-based learning	Questionnaire, quantitative, cross sectional (on overall experience)	Yr 2–4 (*n* = 393), University of Maastricht Medical School, Netherlands	///	unspecified		Lack of effective feedback, lack of effort to find solutions to group conflicts, and lack of motivation were perceived to be the main barriers to assessing professional behaviour. These may be left unaddressed without having negative repercussions to students' academic performances. Unprepared tutors were additionally identified as a concern	
Visschers-Pliejers et al., 2005 [64]	Student perspectives on learning-oriented interactions in the tutorial group	Questionnaire, quantitative, cross sectional (over one course)	Yr 2 (*n* = 175), University of Maastricht Medical School, Netherlands	9	unspecified		Students rated the desirability of learning-oriented interactions (questioning and reasoning) higher than their frequency of occurrence in PBL. Contrastingly, handling conflict was deemed less desirable than its frequency of occurrence	
Papinczak, 2009 [65]	Conducting the symphony: a qualitative study of facilitation in problem-based learning tutorials	Questionnaire, qualitative, cross sectional (on one year)	Yr 1–2 (*n* = 295), University of Queensland Medical School, Australia	8–11	5 h PBL tutorial per week over 36-week school year; PBL involves case analysis, hypotheses formation, and self-directed learning		Students were critical of overly directive or lax tutors, and of tutors who interjected too often or not often enough. Students perceived a greater need for tutors to interject in process-related aspects, such as handling conflict and intervening with dominating personalities. Tutors were viewed as professional role models	
Park et al., 2020 [66]	Shining a light into the black box of group learning: medical students' experiences and perceptions of small groups	Semi-structured interview, qualitative, retrospective on one year	Yr 1 (*n* = 9), UBC Faculty of Medicine, Canada	8	Three 120-min PBL tutorials per week (new case each week) over 6-week units; curriculum is lecture predominant, supplemented by PBL, dissection labs, formal skills training, and portfolio-based mentoring experience; *PBL groups are reassigned every 6 weeks; *tutors are clinicians or health professional or teaching experts		Students understand the intention of PBL to train teamwork skills but preferred self-learning over group learning. Tutors were perceived to be responsible for managing group dynamics, and dysfunctional group behaviour was ignored so long as marks were not impacted. Perception of tutors as an assessor of performance reduced "risk-taking behavior" such as leadership and conflict resolution	
Poskiparta et al., 2003 [67]	Students' and teachers' experiences of a problem-based learning method in health promotion in a Finnish polytechnic	Semi-structured interview, qualitative, retrospective (on one year)	Yr 1–2 nursing students (*n* = 9) and tutors (*n* = 10), unspecified polytechnic, Finland	///	Unspecified, newly implemented PBL curriculum		Students become more favorable of PBL as they become more familiar with the format and more confident with self-direction	
Varga-Atkins et al., 2010 [68]	Developing professionalism through the use of wikis: A study with first-year undergraduate medical students	Questionnaire & focus group, qualitative, cross sectional (on two modules)	Yr 1 (*n* = 32), University of Liverpool School of Medicine, UK	8	Semesters are divided into 2-week modules around PBL cases (week 1 = set learning objectives, then independent study (including wiki use), week 2 = share learning with group)		Good group dynamics and confidence in content knowledge increased likelihood of posting to a shared wiki page. Lack of motivation and fear of making poor-quality contributions decreased engagement. Students were more likely to post to an already active wiki than to start a new one	Engagement with the wiki allowed students to reflect on their identities as knowledge experts and increased their confidence with the material
Hommes et al., 2014 [69]	Understanding the effects of time on collaborative learning processes in problem-based learning: a mixed methods study	Observational, mixed method, longitudinal (over 18 months)	Yr 1–2 (*n* = 173–204), University of Maastricht Medical School, Netherlands	8–12	6–10-week modules; *tutorial groups and tutors are randomly reassigned each module		Groups cohered better over time as students became more familiar with group formation processes. Speed of group coherence was greatly influenced by external factors (e.g., approaching exams)	
Zgheib et al., 2016 [70]	The long-term impact of team-based learning on medical students’ team performance scores and on their peer evaluation scores	Questionnaire, quantitative, longitudinal (over two years)	Yr 1–2 (*n* = 102), American University of Beirut Faculty of Medicine, Lebanon	5–6	Curriculum is half lecture and half non-lecture (TBL, cases, panels, labs); TBL activities include lecture preparation, assigned readings, or a mix of both; TBL learning objectives are given ahead of time; *TBL groups are switched half-way in Yr 1 and again for Yr 2; *students undergo an initial workshop on TBL philosophy and goals		Communication, professionalism, and personal development improved over time. Speed of improvement in teamwork skills also increased with experience	Perceptions of tutorials were generally positive. Students preferred and performed better in tutorials involving lecture preparation as compared to readings-based sessions
Nieminen et al., 2006 [71]	On the relationship between group functioning and study success in problem-based learning	Questionnaire, quantitative, cross sectional (on one PBL session)	Yr 1 medical and dental students (*n* = 116), University of Helsinki, Finland	8–11	Two 90-min PBL tutorials per week over 5-week courses; PBL is supplemented by lectures, clinical sessions, labs, and self-study; students are assessed by an end-of-course test with 3 opportunities to retest		Tutor performance and students’ self-perception of contribution influenced overall experience of group processes	Case quality influenced course grade
Schmidt & Moust, 1995 [72]	What makes a tutor effective? A structural-equations modeling approach to learning in problem-based curricula	Questionnaire, quantitative, cross sectional (over one course)	Yr 1–4 health science students (*n* = 1452), University of Limburg, Netherlands	///	Two 120-min PBL sessions per week over 6-week courses; *PBL groups are randomly assigned		Effective tutors require a combination of content knowledge, the ability to engage with students authentically, and the ability to communicate in accessible language	
Matthew-Maich et al., 2016 [73]	Nursing students' perceptions of effective problem-based learning tutors	Questionnaire, qualitative, cross sectional (on one year)	Yr 1–4 nursing students (*n* = 511), Mohawk College & McMaster University, Canada	10–12	PBL tutorial involves introduction of case scenario, hypothesis formation, identifying learning issues, information gathering and independent study, knowledge debate within group, knowledge application, and reflection of learning process; curricular context is unspecified		Effective tutors were knowledgeable and enthusiastic and inspired motivation to learn in students. In contrast, ineffective tutors were powerful demotivators	
Dolmans et al., 1999 [74]	Is tutor performance dependent on the tutorial group's productivity?: Toward further resolving of inconsistencies in tutor performance	Questionnaire, quantitative, cross sectional (over one module)	Yr 1–4 (*n* = 363 groups), University of Maastricht Medical School, Netherlands	10–12	6-week units		Ratings for tutors with stable performance across tutorials did not vary based on group productivity. Ratings for tutors whose performance was discrepant across tutorials correlated with group productivity	
Ju et al., 2017 [75]	Do medical students generate sound arguments during small group discussions in problem-based learning?: an analysis of preclinical medical students' argumentation according to a framework of hypothetico-deductive reasoning	Observational, quantitative, longitudinal (over one unit)	Yr 1 (*n* = 15), Inje University College of Medicine, Korea	7–8	Three 120-min PBL sessions over first week, followed by 3–4 weeks of lecture per organ block; standardized patients are used in first PBL session			Students were generally poor at providing justification for argumentation during PBL. Most arguments made, regardless of the phase of PBL, were unbacked claims. Fewer claims were backed by data from the case scenario, and even fewer claims were backed by warranted justification
Rotgans et al., 2018 [76]	How cognitive engagement fluctuates during a team-based learning session and how it predicts academic achievement	Questionnaire, quantitative, longitudinal (over one PBL session)	Yr 1–2 (*n* = 175), Lee Kong Chian School of Medicine Singapore, Singapore	5–6	2 TBL sessions per week; both a content expert and a process expert are present; *students are exposed to 3 orientation TBL sessions prior to this study			Regardless of year of study, cognitive engagement was highest during phases of PBL requiring small group interaction and lowest in preparation and class discussion phases
MacLeod, 2011 [77]	Caring, competence and professional identities in medical education	Semi-structured interview, qualitative, retrospective on overall experience	Yr 2 students (*n* = 12) and tutors (*n* = 10), Dalhousie Medical School, Canada	7	unspecified			Various PBL processes allowed students to understand the importance of and display both competence-related (confidence, capability, suitability) and caring-related (benevolence, humbleness) aspects of professional behavior. Gaps in PBL were filled via individual extracurricular engagements

^a^Population is medical students unless otherwise specified

^b^Students are separated by gender for cultural reasons

### Group Composition: Tutor identity

Studies looking at tutor identity compared expert and non-expert tutors, or faculty and student tutors. Student tutors were direct peers from the same cohort, or near-peers, which describes upper years or recent graduates. One study also compared tutored and untutored PBL [30].

Students did not show discriminatory preference (KP1) for content expert or non-expert tutors [31, 32], and appreciated both on different dimensions [33]. Non-experts were valued for group-management skills [31, 34, 35], and experts were rated as better able to navigate discussion around case objectives [34, 35]. However, these KP1 outcomes were not consistently predictive of KP2 learning outcomes [34]. Some studies found expert tutored students scored higher on exams [31, 33], but the majority of the literature reported no significant difference across tutor types in terms of attitudes towards learning and content [34, 36], knowledge and skills acquisition [32, 34, 37–39], and confidence with the material [40]. Similarly, tutors who were more perceived as more socially and cognitively congruent were rated more favorably by students (KP1), but did not produce better recall or module scores (KP2) [41].

Studies comparing faculty to student tutors found a slight preference for the latter (KP1) [34, 37, 40]. Peers and near-peers were rated as better at creating relaxing learning climates [37], more understanding of learner needs [42], more socially congruent [42], and thus more easily accepted by students [40]. Student tutors had greater trouble during case problem discussion and analysis [37, 38], but did not produce worse academic KP2 outcomes than faculty tutors [37, 39, 43].

Tutored PBL was preferred over untutored PBL [30]. On average, untutored students did not perform worse than tutored students on an exam (KP2), though there was greater variability in individual student scores [30].

### Group Composition: Student identity

Students were characterized on dimensions such as baseline achievement and demographic factors.

Student perception of PBL (KP1) were generally favorable and did not significantly increase with achievement [44, 45]. Achievement was variably measured across studies as admission averages, GPA [46], final course grades [39, 44, 45], prior in-class process assessment scores [46, 47], or prior module scores [40, 46]. Overall student achievement appeared to be stable over time [47]. Individual student differences in academic ability [46, 47] and behavior within groups [47] persisted across PBL sessions, suggesting that PBL did not preferentially benefit high or low achieving students over time [47]. However, one study found that feedback improved the quality of student contributions to the group for baseline low-achievers, but not mid- or high-achievers [48].

Demographic diversity such as gender [46, 49, 50], race [49], ethnicity [49], and socioeconomic status [49] did not reliably influence student experience (KP1) or achievement (KP2). Diverse groups attributed a greater importance for diversity, but this had no impact on group function or academic performance [49]. However, the studies displaying these results were primarily conducted in countries with high baseline diversity. Contrastingly, two studies in the Middle East, where students are gender-separated for cultural reasons, found significant gender differences in student perception and behavior [51, 52]. For example, females placed greater priority on acquiring academic knowledge, whereas males valued the opportunity to participate in tutorials [51]. Female groups were also higher in motivation, productivity, and display of positive professional behaviors than male groups [52]. It is thus suggested demographics-related outcomes may be culturally dependent.

Students with more or less PBL experience did not differ in enjoyment of PBL (KP1) or exam scores (KP2) [35]. Some students have also experienced PBL as true-peer or near-peer tutors, which allowed them to facilitate their own cohort or a younger cohort of students, respectively. A systematic review of students with facilitating experiences reported favorable KP1 outcomes such as self-perceived development of professional identity, leadership skills, a sense of confidence and autonomy in their learning, and the ability to reflect on their learning gaps [53]. However, no significant improvements to academic outcomes (KP2) were found [53].

### Group Composition: Other human resource factors

One study examining various PBL groups factors found that groups’ academic performances (KP2) positively correlated to that of its individual members [50]. Both individual and group performance also increased with group size, with the groups ranging from five to seven members [50]. A separate study examining the presence of librarians in PBL tutorials found no impact of the librarian on students attitudes towards PBL (KP1), exam performance (KP2), and confidence in information-searching skills (KP3) [54].

### Group Processes: Student conduct and social climate

Certain student behaviors were favorably perceived (KP1) by students and tutors alike. These centered around themes of respectful assertiveness [55], listening to each other [43, 51, 55], setting clear group goals [55], giving constructive feedback [55, 56], defining and sharing leadership within the group [51, 55], maintaining self-awareness [55, 57], and being prepared for tutorials [55]. Both students and tutors reported good group dynamics and fair allocation of tasks overall [45, 58, 59].

Some favorable behaviors and pleasant group interactions made for enjoyable experiences (KP1) but were not necessarily beneficial to knowledge acquisition (KP2) [55]. For example, contributing to group discussions was viewed by students as important [45, 51, 55], but students who did not actively participate were found to perform equally well on academic assessments [55, 60]. Having group members who were caring or accommodating helped the group feel comfortable, but did not improve learning [55]. Groups may also bond well unproductively, as is the case when students shared a mutual disdain for the learning activity [55].

Contrastingly, unfavorably perceived (KP1) student behaviors included condescension, stubbornness, passivity, and unreliability [55]. Other common student-related issues included quiet and dominant group members [55, 61, 62], lateness and absenteeism [51, 55, 61], rushing through tutorial activities [61], and lack of effective group interactions [61]. Interestingly, both lack of effort for conflict resolution [63] and the act of conflict resolution [64] were perceived by students as undesirable (KP1), in two separate studies.

Similar to the above, unfavorable student behaviors were not linked to worse KP2 outcomes. For instance, several studies found that both students and tutors felt unprepared to deal with conflicts between group members [51, 55, 61, 64, 65], and thus dysfunctional group members and behaviors were most often ignored [51, 63, 66]. Yet, persistent disruptive behaviors were not necessarily harmful to learning or grades (KP2) [55, 61, 66]. In some cases, groups with perceivably poor group dynamics, such as those containing students with aggressive or critical personalities, may even have better KP2 outcomes, as the dominance helps kick-start group discussions and other learning processes [55].

### Group Processes: Motivation and confidence

Studies exploring group processes from a cognitive perspective identified motivation and confidence as factors that effectively map onto experiential (KP1) [32, 44, 53, 67], academic (KP2) [60], and behavioral (KP3) outcomes [53].

Both tutors and students who were more confident in their skills or content knowledge were more likely to engage productively in PBL activities [62, 68] and reported more positive experiences [58, 67]. For example, one study exploring the implementation of a wiki platform for students to share resources found that students with greater confidence in their information-searching skills and understanding of the content were more likely to contribute to the wiki [68]. In several studies, perception of PBL (KP 1), though not learning (KP2), improved slightly with year of study as students became more confident with the process of group formation [69, 70] and with self-direction [32, 51, 67].

Higher motivation and sense of contribution in students mapped onto more favorable KP1 outcomes [67, 71] and higher group productivity [52]. Motivation may be intrinsic, such as pre-existing interest for the content [72], or developed through favorable group interactions [60]. Meaningful group discussions, for example, excited students’ interests toward the content and their desires to learn more [60], while superficial group discussions and unresponsive group members were demotivating and inhibitory to learning (KP2) [60, 63]. As well, tutor qualities may play a role [72, 73]. Students encountered with authentic, passionate, professional, and dependable tutors reported being more incentivized to learn [72, 73], while those who were faced with unprofessional or disengaging tutors reported strongly worded narratives of demotivation from the PBL material, or even the profession at large [73].

### Group Processes: Tutor facilitation

Tutors and students acknowledged that facilitation practice is not standardized [42, 62]. Variations in facilitation practice was not necessarily tied to the level of content expertise of the tutor [33, 42]. Rather, facilitation behaviors were reactionary to the performance of the group [71, 74], the tutor’s perception of their students’ cognitive and practical needs [62], the tutor’s own levels of background knowledge and experience with PBL [34, 58, 62]. For example, some tutors were more willing to actively help resolve student concerns while others favored giving groups the freedom to self-regulate, meaning the nature of group interactions were fundamentally different tutor-to-tutor [62]. Neither content facilitation nor process facilitation consistently produced better PBL outcomes at any KP level [34, 57], though the tutor’s ability to stimulate self-directed learning improved students’ perceptions of PBL case quality and group performance [57].

There exist certain facilitation practices that were generally identified by students as highly problematic in PBL, including both excessively imposing and excessively uninvolved tutors [61, 65, 66] and tutors who were unprepared for tutorial [63]. These views may be moderated by students’ year of study. Students’ facilitation values (KP1) were found to shift slightly over time, where junior students preferred tutors with more content expertise and willingness to provide guidance, and senior students felt a greater need for autonomy from their tutors [73].

Some studies also found that students perceived the need and expectation for tutors to play a greater role in managing group process-related issues, such as dominant or uncommitted students, clashes in student personalities, or lack of group productivity [59, 65, 66]. However, tutors generally felt less prepared to deal with these [61, 62] as compared to content-related issues involving curriculum or case design [61].

Tutors were additionally viewed as role models of professional conduct, such as the handling of sensitive case issues [65].

### PBL Processes: Tutorial activities

Studied PBL processes included a wide range of independent factors such as different natures of tutorial activities [70] and phases [75, 76], group testing [45], knowledge sharing [68], and reflection and feedback [48, 57]. Perceptions of PBL processes were generally favorable across studies [45, 58, 70]. There was good understanding of the intention of PBL to train professional skills, such as teamwork, competence, and caring [66, 77]. Students were able to recognize the importance of these skills in later professional settings [45] and made up for any gaps in PBL curriculum through extracurricular involvements [77]. Engagement with knowledge sharing resources also contributed to students’ exploration of professional identity as knowledge experts and increased their confidence with the content [68].

Some studies suggested students cared a great deal about peer and tutor-given feedback [55, 56]. Providing, reflecting on, and responding to feedback was perceived as helpful for learning and group interaction [55, 56]. In one instance, reflection and feedback on group leadership did not impact leadership behavior, but did increase students’ self-awareness of group roles [56]. Feedback itself was thought to be most effective when received in-person rather than on paper, in the context of clearly defined improvement goals and a willingness to change [48].

Quality of the case-study problems and level of tutorial organization were important to tutors’, but not students’, perceptions of learning effectiveness (KP1) [58]. Case quality was also directly or indirectly important to academic outcomes (KP2) [56, 71]. In one study, tutors reported that case problems, though appropriate for students’ knowledge levels, did not reliably motivate the use of external resources for further learning [58]. Superficial cases and disorganized tutorials were identified in one study as some of the most hindering problems to learning [61].

## Discussion

This review identified several key findings on the role of group function in determining PBL outcomes for students at several KP levels.

Tutor and student demographics did not consistently influence experiential (KP1) or academic (KP2) outcomes. Group diversity did not mark a difference at large, either. This finding may be largely because the nature and degree to which these individuals engaged in a group setting were not significantly influenced by sociodemographic variables [30, 33, 42]. For example, the average expert and non-expert tutors were not different in their teaching strategies, degree of direction, amount and nature of social interaction with students, and ability to utilize their expertise [33, 42]. How the group interacts may have a greater impact on PBL outcome than who the group is composed of, though these interactions were highly context-dependent.

Both extra- and intra-tutorial context has major implications in determining the learning environment in which groups engage. External components in some hybrid PBL curricula, such as concurrent lectures and labs, may confound important outcome variables such as GPA [41]. In the included RCT studies, cross-contamination of students from different tutorials was a frequently reported concern [30, 40, 54]. Within PBL tutorials, aspects of group function may be directly important to learning outcomes or moderate the effects of other aspects. For example, the influence of tutor ability on outcome was moderated by curricular factors (e.g., module structure) [43], characteristics of the group [72, 74], or individual student differences (e.g., comfort with PBL [35], self-study effort [34, 35], innate academic ability [41], and prior knowledge [43]). Facilitation expertise was more important to learning outcome in structurally disorganized than well-organized tutorials [43], in junior than senior students [34], and in students with less experience with PBL [35].

Individual student learning behaviors further complicate the effort to sparse out group function-related outcomes. Length of time engaged in self-study was briefly explored in the literature both as an outcome of group function, either as a favorable indicator of increased interest in the learning [34] or an unfavorable indicator of poor group efficacy [72], and as a confounder to group function-dependent outcomes [71]. For instance, rather than group conflict resolution, self-study was frequently cited as the easier and preferred solution for unsatisfactory teamwork experiences [51, 61, 64, 66]. Dysfunctional group members and processes can thus be left unaddressed, yet remain unharmful to academic KP2 outcomes [51, 55, 63, 66]. Concerns over sustaining individual grades and the pressure to pass exams [62, 69] was suggested to be the cause of bypassing group conflict resolution [66]. This may additionally imply challenges for the ability to measure professionally representative behavioral KP3 outcomes in a PBL classroom [63].

The one factor that seemed to overwrite the situational dependence of learning outcomes appeared to be baseline student achievement, which was consistently linked to several levels of KP outcomes. High-achieving students tended to remain high-achievers throughout their studies, suggesting that some stable factors prevail in successful students regardless of the education process [46, 47]. This may include having more general intrinsic motivation or specific interest in the subject matter [41, 46]. High achievers may also be more confident in their knowledge or have higher self-esteem in academic settings. They may thus find more enjoyment in the learning process, be more motivated to learn, and ultimately perform better, in a positive feedback loop. Indeed, confident students demonstrated higher levels of engagement with PBL activities [68], while students who felt less secure in their social or academic positions were more hesitant to engage in “risk-taking” behavior, such as raise contradictory opinions [75, 76], attempt conflict resolution [66], take on leadership roles [66], and instigate new activities [68]. Interestingly, this extended to tutors as well, such that tutors who were confident in their facilitation skills and training found PBL more enjoyable [58] and were better able to optimize their performance [62]. Thus, it seemed that a sense of social and intellectual safety may be important for all group members to maximize their PBL outcomes at any KP level.

### Limitations of the literature pool

This review identified several limitations of the literature. Most striking was the variability of studies on this topic, which may help explain the vast array of contradictory findings. Some contextual details are provided for each included study in Table 2. Methodologically, the studies were highly different in sample sizes (15 to 9923 participants), response rates (25% to 100%), and studied PBL topic (basic sciences, anatomy, immunology, psychiatry, epidemiology, nursing, etc.). PBL curricula design was also systematically different across institutions. Examples include duration and frequency of tutorials, number of tutorials per case or problem, sizes of the student groups (3 to 21 students), formation of groups (stratified by background or random), degree of student and tutor training, amount of prior PBL experience, and nature and frequency of assessments and feedback. It is expected that these contextual variations in PBL design and implementation, in addition to cultural considerations [78], may complicate results of comprehensive literature reviews of the literature [9]. It is difficult to determine whether these results may be generalized to any singular institution.

A second limitation was lack of reporting. For instance, most studies did not report how groups were formed (*n* = 27), which was notable since group composition influences at least some dimensions of learning outcomes. Though perceptions of PBL were largely favorable, gross curricular details and the range of individual tutorial activities were not well reported by most studies. The effect of these aspects on learning outcome were thus not well characterized.

### Limitations of this review

Finally, this review has limitations as a function of its methodology. This summarization and analysis of the literature is restricted by its chosen frameworks. For example, the KP model is one of many possible lenses of viewing learning outcomes. Other frameworks may include those focusing exclusively on knowledge acquisition [79], identity related outcomes [80], or social outcomes [81], or those that examine outcome at a group rather than individual level. These may all yield uniquely interesting and relevant results in the context of PBL. The definition of PBL in this review may be overly broad in the attempt to be inclusive. A stricter criterion for PBL structure may yield more consistent results. The framework used for group function is a modified version of Fonteijn and Dolmans [25], though other divisions of group function may exist.

## Conclusion

Research on group function in PBL has been broad and diverse, offering a great pool of perspectives on its educational efficacy. This scoping review summarized and structuralized existing literature to provide an organized overview of what aspects of group function are important to individual student outcomes in undergraduate health professional PBL. Such an overview may help educators and researchers navigate this rich field of literature. This review additionally identified the fundamental challenges in linking learning outcomes to group processes, and the gaps where future research may be focused.

The Kirkpatrick framework established experiential (KP1), learning (KP2), and behavioral outcomes (KP3) as independent constructs, which this review found were not reliably correlated to each other in a PBL setting. Key findings showed that student perceptions of PBL and groupwork were generally favorable. Tutor and student demographics did not systematically predict outcomes, and facilitation style and group dynamics were predictors of KP1 but not KP2. Individual student tendencies in KP2 and KP3 persisted. PBL design, such as case quality, tutorial organization, and feedback, were important for KP2 but were not well reported in most studies.

**Fig. 3:**
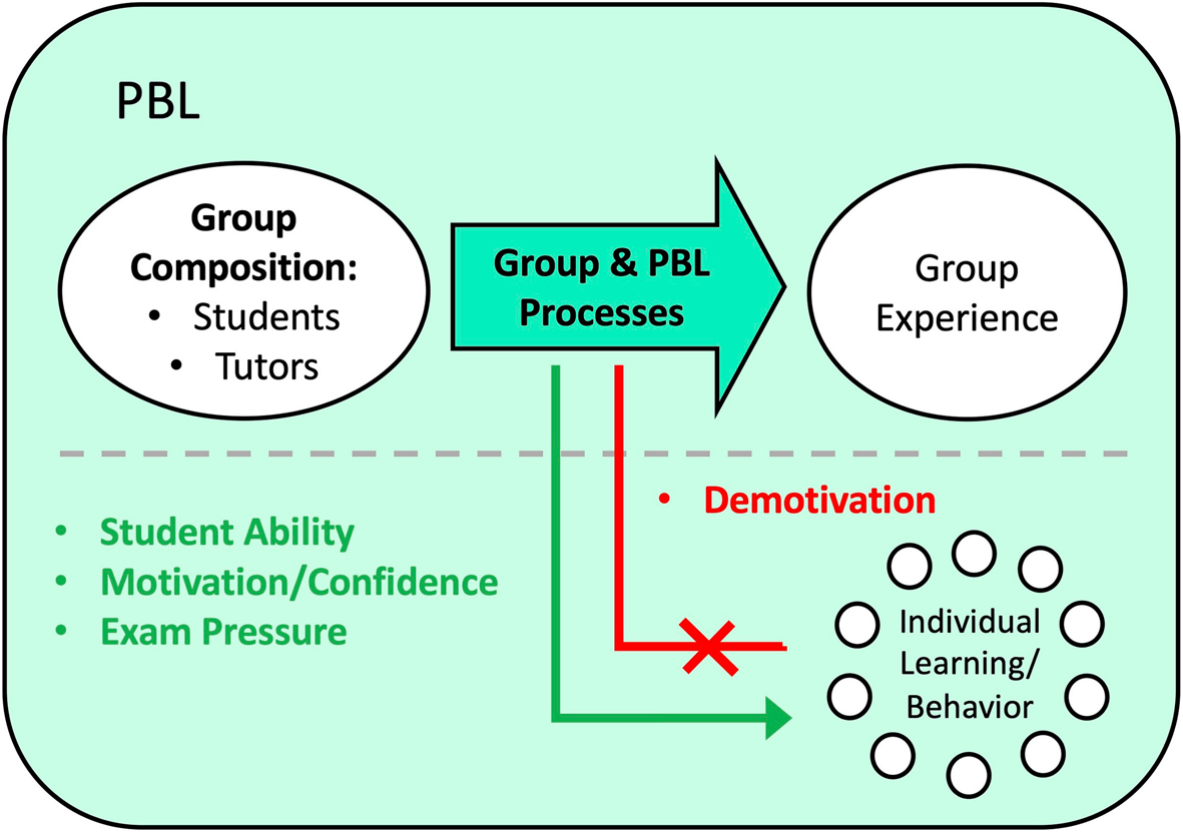
Revised concept of group, PBL, and outcomes to incorporate factors that enhance and impair group and PBL processes

In summary, group function was most directly important to students’ experiences and perceptions of the group and learning (KP1). Knowledge acquisition (KP2) and behavioral (KP3) outcomes were more subject to moderation by stable characteristics in individual students, such as ability, motivation, demotivation, and confidence, as well as external stressors such as exam pressure (Fig. 3).

### Future directions

More research is needed to substantiate the findings of this paper. Reviews of studies at institutions that share similar PBL training and curricula may be required for more conclusive results. Future empirical research is also encouraged to focus on longitudinal behavioral (KP3) outcomes that span the duration of health professional training, or even follow-up into clinical settings, for which there is currently no knowledge. Publications are encouraged to better report the educational, curricular, and training contexts of the institution at which the studies were conducted. Finally, a standardized assessment method for group function in PBL has yet to be developed and may be helpful to future research in this area.

## Supplementary Information

**Additional file 1: Appendix 1. **Search strategy.**  Appendix 2. **Data extraction instrument fields.**  Appendix 3. **Quality appraisal of methodological quality ofincluded studies

## Data Availability

The raw data used and/or analyzed in the current study are available from the corresponding author upon reasonable request.

## Abbreviations

CBL: Case-Based Learning
KP: Kirkpatrick or Kirkpatrick Outcome Level
PBL: Problem-Based Learning
SGL: Small-Group Learning
TBL: Team-Based Learning
