# Supplementary material for: Does your group matter? How group function impacts educational outcomes in problem-based learning: a scoping review
Source: BMC Med Educ. 2022 Dec 29;22:900. doi: 10.1186/s12909-022-03966-8 (PMC9798609; doi:10.1186/s12909-022-03966-8)
Supplement: Supplementary file 1 — Additional file 1: Appendix 1. Search strategy. Appendix 2. Data extraction instrument fields. Appendix 3. Quality appraisal of methodological quality ofincluded studies [file 12909_2022_3966_MOESM1_ESM.docx]

### *Appendix I: Search Strategy*

**Ovid MEDLINE(R) and Epub Ahead of Print, In-Process, In-Data-Review & Other Non-Indexed Citations, Daily and Versions(R) <1946 to January 18, 2022>**

1 exp Education, Medical/ 177062

2 exp Education, Premedical/ 786

3 medical education.mp. [mp=title, abstract, original title, name of substance word, subject heading word, floating sub-heading word, keyword heading word, organism supplementary concept word, protocol supplementary concept word, rare disease supplementary concept word, unique identifier, synonyms] 52703

4 undergraduate medical education.mp. [mp=title, abstract, original title, name of substance word, subject heading word, floating sub-heading word, keyword heading word, organism supplementary concept word, protocol supplementary concept word, rare disease supplementary concept word, unique identifier, synonyms] 3152

5 undergraduate health profession*.mp. [mp=title, abstract, original title, name of substance word, subject heading word, floating sub-heading word, keyword heading word, organism supplementary concept word, protocol supplementary concept word, rare disease supplementary concept word, unique identifier, synonyms] 114

6 PBL.mp. [mp=title, abstract, original title, name of substance word, subject heading word, floating sub-heading word, keyword heading word, organism supplementary concept word, protocol supplementary concept word, rare disease supplementary concept word, unique identifier, synonyms] 9321

7 exp Educational Measurement/ 159797

8 (lifelong learn* or life long learn* or life-long learn*).mp. [mp=title, abstract, original title, name of substance word, subject heading word, floating sub-heading word, keyword heading word, organism supplementary concept word, protocol supplementary concept word, rare disease supplementary concept word, unique identifier, synonyms] 2121

9 learn* outcome.mp. [mp=title, abstract, original title, name of substance word, subject heading word, floating sub-heading word, keyword heading word, organism supplementary concept word, protocol supplementary concept word, rare disease supplementary concept word, unique identifier, synonyms] 489

10 learn* quality.mp. [mp=title, abstract, original title, name of substance word, subject heading word, floating sub-heading word, keyword heading word, organism supplementary concept word, protocol supplementary concept word, rare disease supplementary concept word, unique identifier, synonyms] 98

11 exp Group Processes/ 185843

12 group function*.mp. [mp=title, abstract, original title, name of substance word, subject heading word, floating sub-heading word, keyword heading word, organism supplementary concept word, protocol supplementary concept word, rare disease supplementary concept word, unique identifier, synonyms] 1457

13 group characteristic*.mp. [mp=title, abstract, original title, name of substance word, subject heading word, floating sub-heading word, keyword heading word, organism supplementary concept word, protocol supplementary concept word, rare disease supplementary concept word, unique identifier, synonyms] 773

14 group trait*.mp. [mp=title, abstract, original title, name of substance word, subject heading word, floating sub-heading word, keyword heading word, organism supplementary concept word, protocol supplementary concept word, rare disease supplementary concept word, unique identifier, synonyms] 53

15 11 or 12 or 13 or 14 187978

16 1 or 2 or 3 or 4 or 5 198177

17 7 or 8 or 9 or 10 161737

18 exp Problem-Based Learning/ 9344

19 (problem-base* or problem base*).mp. [mp=title, abstract, original title, name of substance word, subject heading word, floating sub-heading word, keyword heading word, organism supplementary concept word, protocol supplementary concept word, rare disease supplementary concept word, unique identifier, synonyms] 12128

20 6 or 18 or 19 19777

21 15 and 16 and 17 and 20 189

22 limit 21 to (english language and yr="1980 -Current") 185

**CINAHL**

(((MH "Group Processes+")) OR (group function*) OR ("group characteristic*") OR (group trait*)) AND (((MH "Educational Measurement+") OR (MH "Outcomes of Education")) OR ("learn* outcome*") OR (learn* quality)) AND (((MH "Education, Medical+")) OR ((MH "Education, Health Sciences+")) OR ("undergraduate health profession*") OR (undergrad* med*)) AND (pbl OR ("problem based" OR "problem-based") OR ((MM "Problem-Based Learning")))

Limit to (references available, abstract available, english language, 1980-2021)

**APA PsycInfo <1806 to January Week 2 2022>**

1 exp medical education/ 25409

2 exp medical students/ 14068

3 medical education.mp. 21657

4 undergraduate medical education.mp. 611

5 undergraduate health profession*.mp. 35

6 2 or 3 or 4 or 5 28428

7 exp learning/ 283823

8 PBL.mp. 1391

9 (problem-based learn* or problem based learn*).mp. [mp=title, abstract, heading word, table of contents, key concepts, original title, tests & measures, mesh word] 3395

10 (problem-based curricul* or problem based curricul*).mp. [mp=title, abstract, heading word, table of contents, key concepts, original title, tests & measures, mesh word] 97

11 8 or 9 or 10 3684

12 exp group dynamics/ 102674

13 group function*.mp. [mp=title, abstract, heading word, table of contents, key concepts, original title, tests & measures, mesh word] 778

14 group characteristic*.mp. [mp=title, abstract, heading word, table of contents, key concepts, original title, tests & measures, mesh word] 1279

15 group trait*.mp. [mp=title, abstract, heading word, table of contents, key concepts, original title, tests & measures, mesh word] 79

16 12 or 13 or 14 or 15 103627

17 exp educational measurement/ 20244

18 (lifelong learn* or life-long learn* or life long learn*).mp. [mp=title, abstract, heading word, table of contents, key concepts, original title, tests & measures, mesh word] 2487

19 clinical competenc*.mp. 10421

20 learn* outcome.mp. 874

21 learn* quality.mp. 154

22 17 or 18 or 19 or 20 or 21 33815

23 6 and 11 and 16 and 22 10

24 limit 23 to (english language and yr="1980 -Current") 10

###

### *Appendix II: Data Extraction Instrument Fields*

Publication details

- Author(s)
- Title
- Year
- Country
- Study Design
- Qualitative vs Quantitative vs Mixed
- Study take place over: (one PBL session, one unit, one course, one year, multiple years [], retrospective interview on past year/course, retrospective interview on general experience)*
- Cross Sectional vs Longitudinal (Function of Time)*

Participants (If Empirical)

- Study Population
- Years of PBL Experience (Including Current)
- Total Number of Years in Curriculum
- Sample Size (n=)
- Response rate*
- Mean Age (Years)

Studies Included (If Review)

- Inclusion Criteria
- Exclusion Criteria
- Number of Article (n=)

Aims/Purpose

- Was Group Function Part of the Main Study Purpose?: (yes, no)
- Research Question(s) as Stated by Author*
- Is Study Meant to Test a Recent/Potential Changed to Course Structure?: (yes, no)*
- Aspects of Group Function Explored (IV)
- Studied Outcome (DV)
- Kirkpatrick Outcome Level: (1-reaction, 2-learning, 3-behavior, 4-results)

Methodology

- Study methods
- Intervention
- Comparator
- IV Measures
- DV Measures
- Is Scale Used A Validated Scale (Specify)/Part of Course Eval/Other (Specify)?*
- Topic of PBL Session
- PBL Procedure/Structure (i.e., Study Context)
- Group Randomization*
- Number of PBL Sessions Per Case
- Size of PBL Group
- Number of Tutorial Groups

Relevant Key Findings

- Student Identity Related Outcomes (e.g., Years of PBL Experience, High vs Low Achievers)
- Tutor Identity Related Outcomes (e.g., Expert vs Near-Peer)
- Group Interaction Related Outcomes (e.g., Conflict, Frequency of Debate)
- Process Related Outcomes (e.g., Immediate Feedback, Reflections)
- Key Themes

Limitations

- Findings Consistent with Previous Research? (As Identified by Author)
- Limitations (As Identified by Author)
- Missing Information (As Identified by Author)
- Future Directions (As Identified by Author)

Interpreted Relevance of Study

- Well Defined Student Sample?: (yes, somewhat, no)
- Well Defined Tutor Identities?: (yes, somewhat, no)
- Well Defined PBL process?: (yes, somewhat, no)
- PBL Context Given?: (yes, no)*

Notes

### *Appendix III: Quality Appraisal of Methodological Quality of Included Studies*

1. **Appraisal of Quantitative and Mixed-Methods Studies (MERSQI Tool)**

| **Citation: Author,**  **Year** | **Study Design** | **Sampling: Institutions** | **Sampling: Response Rate** | **Type of Data** | **Validity of Evaluation Instrument** | **Data Analysis: Sophistication** | **Data Analysis: Appropriate** | **Outcome** | Total Score |
| --- | --- | --- | --- | --- | --- | --- | --- | --- | --- |
| Hayashi et al., 2013^30^ | 3 | 0.5 | 0.5 | 3 | 0 | 2 | 1 | 1.5 | **11.5** |
| Hay & Katsikitis, 2001^31^ | 3 | 0.5 | 0.5 | 3 | 2 | 2 | 1 | 1.5 | **13.5** |
| Shields et al., 2007^32^ | 1.5 | 0.5 | 1.5 | 3 | 0 | 2 | 1 | 1.5 | **11** |
| Davis et al., 1992^33^ | 1 | 0.5 | 1.5 | 3 | 0 | 1 | 1 | 1.5 | **9.5** |
| Schmidt et al., 1993^34^ | 1 | 0.5 | 0.5 | 3 | 2 | 2 | 1 | 1.5 | **11.5** |
| Qin et al., 2010^35^ | 1 | 0.5 | 1.5 | 3 | 0 | 1 | 1 | 1.5 | **9.5** |
| Groves et al., 2005^36^ | 1 | 0.5 | 1.5 | 1 | 2 | 2 | 1 | 1 | **10** |
| Kassab et al., 2005^37^ | 3 | 0.5 | 1 | 3 | 1 | 2 | 1 | 1.5 | **13** |
| Kassab et al., 2005^38^ | 3 | 0.5 | 1 | 3 | 1 | 2 | 1 | 1.5 | **13** |
| Ten Cate et al., 2012^39^ | 1 | 0.5 | 0.5 | 3 | 2 | 2 | 1 | 1.5 | **11.5** |
| Widyahening et al., 2019^40^ | 3 | 0.5 | 1.5 | 3 | 2 | 2 | 1 | 1.5 | **14.5** |
| Chng et al., 2015^41^ | 1 | 0.5 | 1.5 | 3 | 2 | 2 | 1 | 1.5 | **12.5** |
| Schmidt, 1994^43^ | 1 | 0.5 | 1 | 3 | 0 | 2 | 1 | 1.5 | **10** |
| Vasan et al., 2009^44^ | 1 | 0.5 | 1.5 | 1 | 2 | 2 | 1 | 1 | **10** |
| Gallagher, 2009^45^ | 1 | 0.5 | 0.5 | 1 | 1 | 1 | 1 | 1 | **7** |
| Wahid et al., 2015^46^ | 1 | 0.5 | 1 | 3 | 2 | 2 | 1 | 1.5 | **12** |
| Wimmers & Lee, 2015^47^ | 1 | 0.5 | 1.5 | 3 | 2 | 2 | 1 | 1.5 | **12.5** |
| Kamp et al., 2013^48^ | 1.5 | 0.5 | 1.5 | 1 | 1 | 2 | 1 | 1.5 | **10** |
| Ganguly et al., 2019^49^ | 1 | 0.5 | 1.5 | 3 | 0 | 2 | 1 | 1.5 | **10.5** |
| Thompson et al., 2015^50^ | 1 | 1.5 | 1.5 | 3 | 2 | 2 | 1 | 1.5 | **13.5** |
| Mpofu et al., 1998^51^ | 1 | 0.5 | 1.5 | 1 | 1 | 2 | 1 | 1 | **9** |
| Das Carlo et al., 2003^52^ | 1 | 0.5 | 1.5 | 3 | 2 | 2 | 1 | 1.5 | **12.5** |
| Koufogiannakis et al., 2005^54^ | 3 | 0.5 | 0.5 | 3 | 1 | 2 | 1 | 1.5 | **12.5** |
| Van Berkel & Dolmans, 2006^56^ | 1 | 0.5 | 1 | 3 | 2 | 2 | 1 | 1.5 | **12** |
| Alizadeh et al., 2017^57^ | 1.5 | 0.5 | 1.5 | 3 | 2 | 2 | 1 | 1.5 | **13** |
| Kingsbury & Lymn, 2008^58^ | 1 | 0.5 | 0.5 | 1 | 1 | 2 | 1 | 1 | **8** |
| Dolmans et al., 2001^59^ | 1 | 0.5 | 1.5 | 1 | 2 | 2 | 1 | 1 | **10** |
| Hendry et al., 2003^61^ | 1 | 0.5 | 0.5 | 1 | 0 | 1 | 1 | 1 | **6** |
| ODoherty et al., 2018^62^ | 1 | 0.5 | 1.5 | 1 | 1 | 1 | 1 | 1 | **8** |
| Van Mook et al., 2007^63^ | 1 | 0.5 | 1 | 1 | 2 | 2 | 1 | 1 | **9.5** |
| Visschers-Pliejers et al., 2005^64^ | 1 | 0.5 | 1 | 1 | 2 | 2 | 1 | 1 | **9.5** |
| Hommes et al., 2014^69^ | 1 | 0.5 | 1.5 | 1 | 1 | 2 | 1 | 1 | **9** |
| Zgheib et al., 2016^70^ | 1 | 0.5 | 1.5 | 3 | 3 | 2 | 1 | 1.5 | **13.5** |
| Nieminen et al., 2006^71^ | 1 | 0.5 | 1.5 | 3 | 2 | 2 | 1 | 1.5 | **12.5** |
| Schmidt & Moust, 1995^72^ | 1 | 0.5 | 1.5 | 3 | 2 | 2 | 1 | 1.5 | **12.5** |
| Dolmans et al., 1999^74^ | 1 | 0.5 | 0.5 | 1 | 1 | 2 | 1 | 1 | **8** |
| Ju et al., 2017^75^ | 1 | 0.5 | n/a | 3 | 2 | 1 | 1 | 1.5 | **10** |
| Rotgans et al., 2018^76^ | 1 | 0.5 | 0.5 | 3 | 2 | 2 | 1 | 1.5 | **11.5** |

1. **Appraisal of Qualitative Studies (Côté and Turgeon Grid)**

| **Citation: Author,**  **Year** | **Introduction: The issue is described clearly and corresponds to the current state of knowledge.** | **Introduction: The research question and objectives are clearly stated and are relevant to qualitative research.** | **Methods: The context of the study and the researchers’ roles are clearly described.** | **Method: The method is appropriate for the research question.** | **Method: The selection of participants is appropriate to the research question and to the method selected.** | **Method: The process for collecting data is clear and relevant.** | **Method: Data analysis is credible.** | **Results: The main results are presented clearly.** | **Results: The quotations make it easier to understand the results.** | **Discussion: The results are interpreted in credible and innovative ways.** | **Discussion: The limitations of the study are presented.** | **Conclusion: The conclusion presents a synthesis of the study and proposes avenues for further research.** |
| --- | --- | --- | --- | --- | --- | --- | --- | --- | --- | --- | --- | --- |
| Cianciolo et al., 2016^42^ | **✓** | **✓** | ± | ± | **✓** | **✓** | **✓** | **✓** | **✓** | **✓** | **✓** | **✓** |
| Iqbal et al., 2016^55^ | **✓** | **✓** | ± | **✓** | ± | **✓** | **✓** | ± | **✓** | **✓** | **✓** | **✓** |
| Papinczak, 2009^65^ | **✓** | **✓** | ± | ± | ± | ± | **✓** | **✓** | **✓** | ± | **✓** | **✓** |
| Park et al., 2020^66^ | **✓** | ± | ± | **✓** | **✓** | **✓** | ± | **✓** | **✓** | **✓** | **✓** | **✓** |
| Poskiparta et al., 2003^67^ | **✓** | **✓** | **✗** | **✓** | ± | ± | ± | **✓** | ± | **✓** | **✓** | ± |
| Varga-Atkins et al., 2010^68^ | **✓** | **✓** | **✗** | **✓** | **✓** | ± | **✓** | **✓** | **✓** | **✓** | ± | **✓** |
| Matthew-Maich et al., 2016^73^ | **✓** | **✓** | ± | **✓** | **✓** | **✓** | **✓** | **✓** | ± | **✓** | ± | ± |
| MacLeod, 2011^77^ | **✓** | **✓** | **✗** | **✓** | ± | **✓** | ± | **✓** | **✓** | **✓** | ± | **✗** |
| **✓= Yes** ±**= Somewhat ✗= No** | | | | | | | | | | | | |

1. **Appraisal of Systematic and Scoping Reviews (JBI Checklist)**

| **Citation: Author,**  **Year** | **Is the review question clearly and explicitly stated?** | **Were the inclusion criteria appropriate for the review question?** | **Was the search strategy appropriate?** | **Were the sources and resources used to search for studies adequate?** | **Were the criteria for appraising studies appropriate?** | **Was critical appraisal conducted by two or more reviewers independently?** | **Were there methods to minimize errors in data extraction?** | **Were the methods used to combine studies appropriate?** | **Was the likelihood of publication bias assessed?** | **Were recommendations for policy and/or practice supported by the reported data?** | **Were the specific directives for new research appropriate?** |
| --- | --- | --- | --- | --- | --- | --- | --- | --- | --- | --- | --- |
| Burgess et. al., 2014^53^ | Yes | Unclear | Yes | Unclear | n/a | No | Yes | Yes | n/a | Yes | Yes |
| Dolmans & Schmidt, 2006^60^ | Yes | Yes | No | No | n/a | No | No | Yes | n/a | Yes | Yes |
